# Development of a complete blood count with differential—based prediction model for in-hospital mortality among patients with acute myocardial infarction in the coronary care unit

**DOI:** 10.3389/fcvm.2022.1001356

**Published:** 2022-10-05

**Authors:** Yu Wang, Changfu Li, Miao Yuan, Bincheng Ren, Chang Liu, Jiawei Zheng, Zehao Lin, Fuxian Ren, Dengfeng Gao

**Affiliations:** ^1^Department of Cardiology, Xi’an Jiaotong University Second Affiliated Hospital, Xi’an, China; ^2^Department of Digestive Medicine, Daqing Longnan Hospital, Daqing, China; ^3^Department of Cardiology, Meishan Branch of the Third Affiliated Hospital, Yanan University School of Medical, Meishan, China

**Keywords:** complete blood count with differential, prediction model, mortality, acute myocardial infarction, coronary care unit, neural network

## Abstract

**Purpose:**

In recent years, the complete blood count with differential (CBC w/diff) test has drawn strong interest because of its prognostic value in cardiovascular diseases. We aimed to develop a CBC w/diff-based prediction model for in-hospital mortality among patients with severe acute myocardial infarction (AMI) in the coronary care unit (CCU).

**Materials and methods:**

This single-center retrospective study used data from a public database. The neural network method was applied. The performance of the model was assessed by discrimination and calibration. The discrimination performance of our model was compared to that of seven other classical machine learning models and five well-studied CBC w/diff clinical indicators. Finally, a permutation test was applied to evaluate the importance rank of the predictor variables.

**Results:**

A total of 2,231 patient medical records were included. With a mean area under the curve (AUC) of 0.788 [95% confidence interval (CI), 0.736–0.838], our model outperformed all other models and indices. Furthermore, it performed well in calibration. Finally, the top three predictors were white blood cell count (WBC), red blood cell distribution width-coefficient of variation (RDW-CV), and neutrophil percentage. Surprisingly, after dropping seven variables with poor prediction values, the AUC of our model increased to 0.812 (95% CI, 0.762–0.859) (*P* < 0.05).

**Conclusion:**

We used a neural network method to develop a risk prediction model for in-hospital mortality among patients with AMI in the CCU based on the CBC w/diff test, which performed well and would aid in early clinical decision-making. The top three important predictors were WBC, RDW-CV and neutrophil percentage.

## Introduction

In recent decades, early reperfusion therapy and adjunctive pharmacotherapy have improved the outcomes of acute myocardial infarction (AMI) (1). Globally, in-hospital mortality has decreased from 29% in 1969 (2) to approximately 5% today (3, 4). However, the contemporary in-hospital mortality varies substantially across patients in different risk groups. Risk stratification in patients with AMI is important for clinical decision-making. Traditional risk score systems mostly derive from ideal clinical trials with stringent patient cohort selection (5–7). We aimed to develop and assess a risk prediction model in a real-world cohort.

The complete blood count with differential (CBC w/diff) test provide a wealth of information on the inflammatory state, oxygen-carrying capacity, and coagulation state. Many indices are accepted prognostic markers for short- and long-term outcomes following AMI, such as white blood cell count (WBC) (8), neutrophil to lymphocyte ratio (NLR) (9), platelet to lymphocyte ratio (PLR) (10), systemic immune-inflammation index (SII, calculated as *P* × *N*/*L*, where *P* and *N*/*L* are the absolute platelet count (PLT) and the NLR, respectively) (11), and red cell distribution width (RDW) (12). The CBC w/diff indices have drawn strong interest because of their low-cost and easy availability in clinical practice. However, existing cardiovascular prognostic scoring systems do not include predictors derived from the CBC w/diff indices. Moreover, it is unclear whether a comprehensive model incorporating all of these indices would have better predictive ability, and which index is the most powerful predictor among them has yet to be identified. Hence, we used all of these indices to create a prediction model and ranked the predictive values in order of importance to the model.

Traditional regression models are constrained by a failure to account for non-linear effects and complex interactions among predictor variables (3). In this study, we developed a prediction model based on the neural network method. Our model’s prediction performance was then compared to other models developed using the most recently published classical machine learning methods, including traditional logistic regression (LR), k-nearest neighbor (KNN), support vector machine (SVM), Gaussian naive Bayes (GNB), adaptive boosting (AdaBoost), decision tree (DT), and random forest (RF).

We aimed to develop a CBC w/diff-based neural network prediction model for in-hospital mortality among patients with severe AMI in the CCU, as well as to identify the most important predictor variable in the CBC w/diff test.

## Materials and methods

### Design, data source, and ethical statement

This current study was a single-center retrospective study. We used the clinical information of patients from a public database to develop a prediction model for in-hospital mortality.

We obtained data from a freely available public database called the Medical Information Mart for Intensive Care-IV (MIMIC-IV, Published: March 16th, 2021. Version: 1.0)^1^ (13). This database contains de-identified clinical data of a large number of patients admitted to the Beth Israel Deaconess Medical Center (BIDMC, Boston, MA, USA) from 2008 to 2019 (inclusive). One of the authors gained access to the dataset by completing the Collaborative Institutional Training Initiative program course (Certificate Record ID: 39690061).

Informed consent was waived for this study because of the perfect anonymous data. We conducted this study in accordance with the Declaration of Helsinki. We report this study according to the Transparent Reporting of multivariable prediction model for Individual Prognosis or Diagnosis (TRIPOD) guidelines (14).

### Patient selection

**FIGURE 1:**
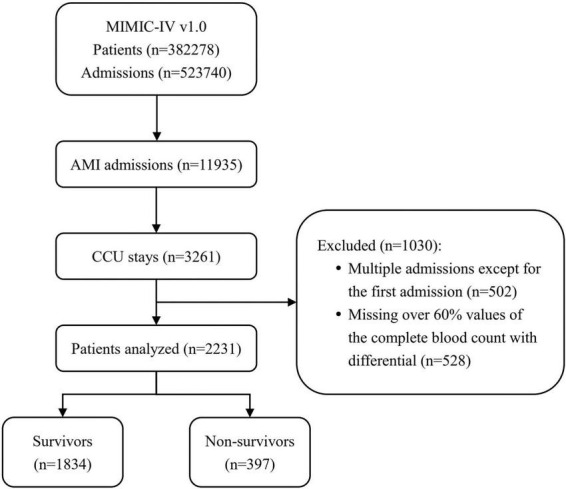
Flowchart of patient inclusion. The medical information mart for intensive care IV (MIMIC-IV) (version 1.0) database includes medical records of 5,23,740 admissions from 3,82,278 patients. In total, 11,935 admissions had a discharge diagnosis of acute myocardial infarction (AMI), of which 3,261 were admitted to the coronary care unit (CCU). After excluding 502 multiple admissions except for the first admission and 528 patients missing over 60% values of the complete blood count with differential (CBC w/diff) test, 2,231 patients were ultimately included in this study, of whom 397 died. MIMIC, medical information mart for intensive care; AMI, acute myocardial infarction; CCU, coronary care unit; CBC w/diff, complete blood count with differential.

In the initial inclusion cohort, all patients with International Classification of Diseases, 9th revision (ICD - 9) codes of the 410 groups or ICD, 10th revision (ICD-10) codes of the I21 groups (the ICD codes of AMI) were included on discharge. Then, those admitted to the CCU were chosen from these patients. To improve comparability among patients, only initial admission data were used for patients admitted to the hospital more than once. Finally, patients who had a CBC w/diff test with more than 60% missing values were excluded. Figure 1 shows the details of the selection process used in this study.

### Data extraction

We extracted the data using structure query language (SQL) with PostgreSQL software (version 13) and Navicat Premium software (version 15). The code that supports the MIMIC-IV is publicly available.^2^ In addition to the parameters in CBC w/diff at admission, we also extracted the baseline characteristics related to patients’ cardiovascular risks, including demographics, comorbidities, laboratory values, and other clinical parameters. The outcome of interest was all-cause in-hospital death marked in the electronic medical record system on discharge. We did not calculate the sample size because all selected data in the database were used to maximize the generalizability and power of the findings.

### Missing values

Missing values were imputed by the missForest method (15), which has been proven to outperform all other algorithms. After completing the missing values, we normalized the 19 variables by column to facilitate subsequent data analysis.

### Model development

**FIGURE 2:**
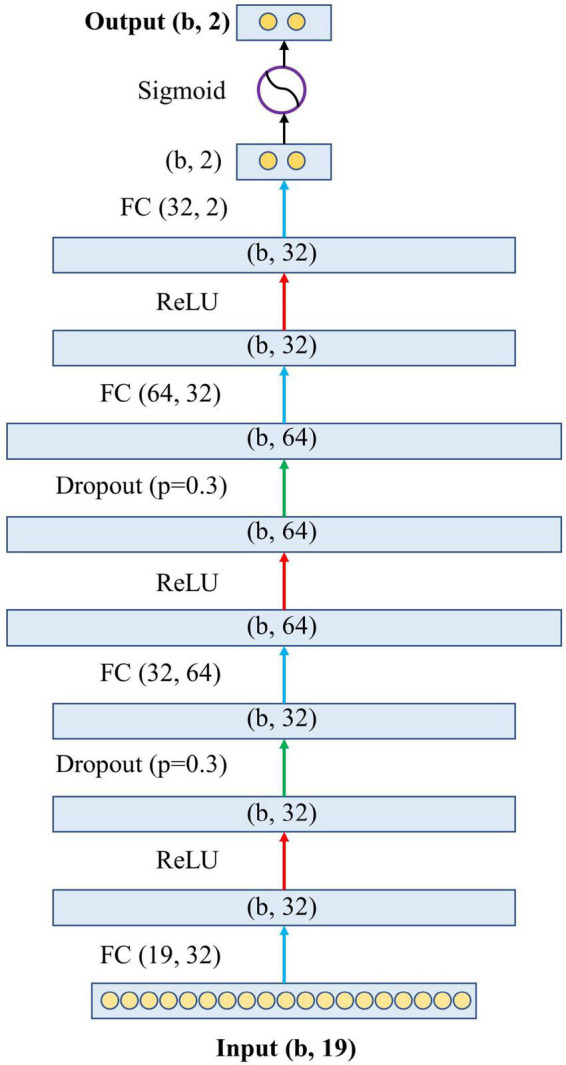
The architecture of the proposed neural network. The letter “b” in the figure represents the batch size of the model, which is the number of samples used to train a single forward and backward pass. FC, fully connected layer; ReLU, rectified linear unit.

Figure 2 shows the architecture of the proposed model. Specifically, the proposed model has one input layer, one output layer, and four hidden layers in between. The input layer contains 19 neurons, representing the 19 variables in the blood test. The output layer contains two neurons representing the predicted probabilities of the model for survival and death, and the sum of these two probabilities is 1. The four hidden layers have feature dimensions of 32, 64, 32, and 2. This design simulates the complex relationship between variables and outcomes by allowing the 19 variables to undergo sufficient non-linear transformation. The hidden layers are connected by the activation function Rectified Linear Unit (ReLU) (16) and the dropout layer (17). Finally, before the output layer, there is a Sigmoid function (18) to generate the final association outcome. The Sigmoid function was chosen because it shrank the final output values into an S-shaped curve ranging from 0 to 1, representing the probability value predicted by the model.

Our neural network was built using PyTorch (version 1.1.2). We chose stochastic gradient descent (19) as the optimizer. The weighted cross-entropy (20) was used as the loss function, where the weight of negative samples was set to 0.2, and the weight of positive samples was set to 0.8. This weight set was created because the number of negative samples is roughly four times that of positive samples. In addition, the relevant hyperparameter settings were as follows: epoch was set to 200, batch size was 32, learning rate was 0.001, and momentum was 0.9.

The model’s final performance was determined by taking the average of the results of the 5-fold cross-validation, which is currently a preferred technique in computer science.

### Statistical analysis

Two-sided *P*-values ≤ 0.05 were considered statistically significant. Baseline characteristics analyses were conducted using SPSS software (version 23). Other statistical analyses were calculated and plotted using Python (version 3.7.5).

#### Comparison of baseline characteristics

The baseline characteristics of patients were compared between survivors and non-survivors. The Kolmogorov–Smirnov test was used to assess the normality of the distribution. Data are presented as the mean with standard deviation for normally distributed continuous variables or median with interquartile range for skewed data. Independent sample *t*-tests or Mann–Whitney U tests were used as appropriate. Categorical variables are presented as absolute numbers with percentages and were compared by using the chi-square test.

#### Model assessment

The performance of our newly developed model was evaluated using discrimination and calibration on the testing set in accordance with the guidelines of the Discrimination and Calibration of Clinical Prediction Models (21). Our model’s discrimination performance was compared to seven classical machine learning models (including LR, KNN, SVM, GNB, AdaBoost, DT, and RF) and five well-studied CBC w/diff clinical indicators (including WBC, RDW-CV, NLR, PLR, and SII). WBC was the whole white blood cell count. RDW-CV was the red blood cell distribution width-coefficient of variation in this study. The NLR was the ratio of the absolute count or percentage of neutrophils to lymphocytes. The PLR was the absolute platelet to lymphocyte count ratio. The SII was calculated as *P* × *N*/*L*, where *P* was the absolute PLT and *N*/*L* was the NLR. To evaluate discrimination performance, receiver operating characteristic (ROC) curves were plotted. The area under the curve (AUC) with 95% confidence interval (CI), sensitivity, specificity, accuracy (ACC), precision, and *F*1-score were all calculated. DeLong’s test was used to assess the difference in AUC between our model and another model or index. To demonstrate the calibration performance, a calibration curve was constructed. Given that the visual representation of the correlation between predicted and observed values is sufficient to evaluate calibration performance and that statistical tests such as the Hosmer–Lemeshow test have limitations (21), we did not test for significant differences. We also calculated the Brier score. The Brier score offers a more comprehensive assessment of model performance, combining model discrimination and calibration. The Brier score represents the mean squared difference between the predictions and the observed outcome. When two models are compared, a smaller Brier score indicates better model performance.

### Feature importance rank

This study applied a permutation test to evaluate the importance rank of features (22). According to permutation test theory, a feature is considered important if the model’s prediction error increases after permuting its values, indicating that the model’s predictive capability is heavily reliant on that feature. The importance of a feature in our model was determined by its effect on ACC and AUC. A feature was considered important if the sum of ACC and AUC deteriorated significantly as a result of the permuting process.

## Results

### Baseline characteristics of the study population

According to the study objective, we included medical records from the MIMIC-IV database of patients diagnosed with AMI who were admitted to the CCU for the first time. After the selection process, 2,759 records were found to be eligible, but 528 of them had incomplete data, with more than 60% of the CBC w/diff values missing. Ultimately, a total of 2,231 medical records were included in this study. We compared the baseline characteristics of patients who were included and those who were excluded due to missing data (Table 1). There was no statistically significant difference between the two groups in terms of age, sex, or body mass index (BMI). Notably, patients in the included group had a higher rate of comorbidities and in-hospital mortality than patients in the excluded group. This study aimed to mine data from the severe population in the intensive care records database, so mortality was markedly higher than in other studies in general medical centers.

**TABLE 1:** Comparison of baseline clinical characteristics between included and excluded patients.

Characteristics	Included (*n* = 2231)	Excluded (*n* = 528)	*P*-value
Age (years)	72.25 (62.92–81.60)	71.83 (60.04–88.98)	0.420
Sex [male, *n* (%)]	1378 (61.8%)	316 (59.8%)	0.416
BMI (kg/m^2^)	28.85 (26.33–29.98)	28.85 (27.27–28.95)	0.816
Hypertension [*n* (%)]	865 (38.8%)	234 (44.3%)	**0.019**
Cardiac arrhythmia			
Cardiac arrest [*n* (%)]	214 (9.5%)	32 (6.1%)	**0.010**
Atrial fibrillation [*n* (%)]	745 (33.4%)	133 (25.2%)	**<0.001**
Congestive heart failure [*n* (%)]	1398 (62.7%)	225 (42.6%)	**<0.001**
Peripheral vascular disease [*n* (%)]	358 (16.0%)	79 (15.0%)	0.539
Cerebrovascular disease [*n* (%)]	247 (11.1%)	37 (7.0%)	**0.006**
Diabetes mellitus [*n* (%)]	934 (41.9%)	166 (31.4%)	**<0.001**
In-hospital death [*n* (%)]	397 (17.8%)	66 (12.5%)	**<0.001**

*P* ≤ 0.05 are indicated in bold.

BMI, body mass index.

Table 2 shows the baseline characteristics of the 2,231 patients who were included in the study. Among the 2,231 patients (median age, 72.2 [62.9–81.6] years; sex, 1,378 [61.8%] male), 397 [17.8%] died in the hospital. The characteristics of survivors and non-survivors were compared. The non-survival group had a higher age, more comorbidities, higher blood creatinine, higher blood glucose, a faster heart rate, and lower blood pressure than the survival group.

**TABLE 2:** Comparison of baseline clinical characteristics between survivors and non-survivors in the included cohort.

Characteristics	All patients (*n* = 2231)	Survivors (*n* = 1834)	Non-survivors (*n* = 397)	*P*-value
**Demographics**				
Age (years)	72.2 (62.9–81.6)	71.1 (62.0–80.6)	77.1 (67.6–84.7)	**<0.001**
Sex [male, *n* (%)]	1378 (61.8%)	1147 (62.5%)	231 (58.2%)	0.105
**Comorbidities**				
Hypertension [*n* (%)]	865 (38.8%)	759 (41.4%)	106 (26.7%)	**<0.001**
Diabetes mellitus [*n* (%)]	934 (41.9%)	750 (40.9%)	184 (46.3%)	**0.046**
Peripheral vascular disease [*n* (%)]	358 (16.0%)	278 (15.2%)	80 (20.2%)	**0.014**
Cerebrovascular disease [*n* (%)]	247 (11.1%)	180 (9.8%)	67 (16.9%)	**<0.001**
Congestive heart failure [*n* (%)]	1398 (62.7%)	1117 (60.9%)	281 (70.8%)	**<0.001**
Cardiac arrhythmia				
Atrial fibrillation [*n* (%)]	745 (33.4%)	566 (30.9%)	179 (45.1%)	**<0.001**
Cardiac arrest [*n* (%)]	214 (9.6%)	112 (6.1%)	102 (25.7%)	**<0.001**
**Laboratory variables**				
Troponin T (ng/mL)	1.22 (0.32–2.45)	1.18 (0.32–2.45)	1.36 (0.33–2.46)	0.397
CKMB (ng/mL)	35 (9–67)	35 (9–67)	36 (9–67)	0.669
Creatinine (mg/dL)	1.2 (0.9–1.8)	1.1 (0.9–1.6)	1.6 (1.2–2.4)	**<0.001**
Glucose (mg/dL)	141 (113–196)	137 (112–186)	171 (121–243)	**<0.001**
**Clinical parameters**				
BMI (kg/m^2^)	28.9 (26.3–30.0)	28.9 (26.6–30.0)	28.9 (25.3–30.1)	0.067
Heart rate (bpm)	86 (73–95)	85 (73–94)	88 (76–103)	**<0.001**
SBP (mmHg)	123 (109–135)	123 (111–137)	117 (101–126)	**<0.001**
DBP (mmHg)	70 (60–78)	70 (61–79)	67 (55–76)	**<0.001**
SpO_2_ (%)	97 (95–99)	97 (95–99)	96 (94–100)	0.849

*P* ≤ 0.05 are indicated in bold.

CKMB, creatine kinase - MB isoenzyme; BMI, body mass index; SBP, systolic blood pressure; DBP, diastolic blood pressure; SpO_2_, pulse oxygen saturation.

The majority of predictor variables in the CBC w/diff test at admission were included in our newly developed model. Table 3 shows the levels of each variable in all patients, as well as in the survival and non-survival groups. The values of the majority of variables differed significantly between the survival and non-survival groups.

**TABLE 3:** Comparison of baseline complete blood count with differential characteristics between survivors and non-survivors in the included cohort.

Characteristics	All patients (*n* = 2231)	Survivors (*n* = 1834)	Non-survivors (*n* = 397)	*P*-value
WBC (K/μL)	11.10 (8.30–14.50)	10.90 (8.30–14.00)	12.60 (8.75–17.40)	**<0.001**
RBC (m/uL)	3.88 (3.38–4.36)	3.88 (3.43–4.42)	3.67 (3.20–4.10)	**<0.001**
Hemoglobin (g/dL)	11.6 (10.0–13.1)	11.6 (10.2–13.2)	10.9 (9.5–12.2)	**<0.001**
Platelet count (K/μL)	217 (169–269)	218 (172–269)	207 (151–270)	**0.022**
Neutrophil percentage (%)	79.9 (70.4–86.0)	79.2 (69.7–85.4)	82.6 (74.6–87.5)	**<0.001**
Monocyte percentage (%)	5.4 (3.7–7.5)	5.4 (3.8–7.5)	5.1 (3.1–7.9)	0.460
Lymphocyte percentage (%)	12.1 (7.3–19.7)	13.0 (7.9–20.5)	9.0 (5.8–15.0)	**<0.001**
Eosinophil percentage (%)	0.6 (0.1–1.7)	0.6 (0.2–1.8)	0.2 (0–1.0)	**<0.001**
Basophil percentage (%)	0.3 (0.2–0.5)	0.3 (0.2–0.5)	0.2 (0.1–0.4)	**<0.001**
Neutrophil count (K/μL)	13.05 (7.45–742.06)	11.64 (6.86–733.21)	16.14 (9.25–861.23)	**<0.001**
Monocyte count (K/μL)	23.46 (0.83–49.78)	26.79 (0.85–50.42)	1.96 (0.78–47.10)	**0.004**
Lymphocyte count (K/μL)	64.38 (1.39–147.92)	73.63 (1.51–155.19)	3.61 (1.01–112.11)	**<0.001**
Eosinophil count (K/μL)	1.21 (0.03–9.00)	1.66 (0.05–10.38)	0.14 (0.01–3.97)	**<0.001**
Basophil count (K/μL)	0.59 (0.03–3.69)	1.10 (0.03–3.91)	0.06 (0.01–2.22)	**<0.001**
Hematocrit (%)	35.5 (30.7–39.4)	35.2 (30.9–39.7)	33.7 (29.8–37.9)	**<0.001**
MCV (fL)	91.1 (87.0–95.0)	91.1 (87.0–94.0)	93.0 (88.0–97.0)	**<0.001**
MCH (pg)	30.0 (28.8–31.3)	30.0 (28.9–31.3)	29.9 (28.4–31.4)	0.177
MCHC (%)	32.9 (32.0–33.9)	32.9 (32.2–34.0)	32.3 (31.1–33.2)	**<0.001**
RDW-CV (%)	14.3 (13.4–15.2)	14.1 (13.3–14.9)	14.8 (13.9–16.6)	**<0.001**

*P* ≤ 0.05 are indicated in bold.

CBC w/diff, complete blood count with differential; WBC, white blood cell; RBC, red blood cell; MCV, mean corpuscular volume; MCH, mean corpuscular hemoglobin; MCHC, mean corpuscular hemoglobin concentration; RDW-CV, red cell distribution width-coefficient of variation.

### The discrimination performance of models and clinical indices

**FIGURE 3:**
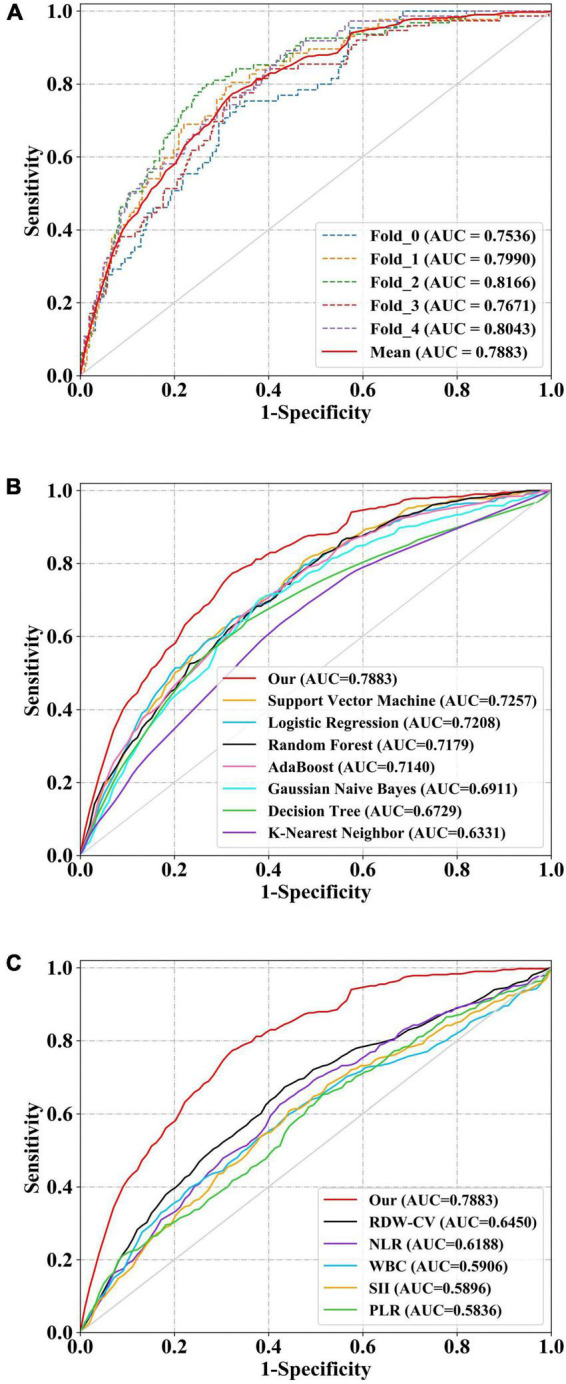
Receiver operator characteristic (ROC) curves of models and indices for predicting in-hospital mortality. The solid red line indicates the mean ROC curve of our neural network model. **(A)** The solid red line indicates the average result of the 5-fold cross-validation. The other five dotted lines represent the result of each fold. **(B)** Our model had a significantly higher mean area under the curve (AUC) of 0.788 (95% CI, 0.736–0.838) than other models [including logistic regression (LR), k-nearest neighbor (KNN), support vector machine (SVM), Gaussian naive Bayes (GNB), adaptive boosting (AdaBoost), decision tree (DT), and random forest (RF)] (*P* < 0.05). **(C)** The AUC of our model also outperformed that of other indices [includingwhite blood cell count (WBC), neutrophil-to-lymphocyte ratio (NLR), platelet to lymphocyte ratio (PLR), systemic immune-inflammation index (SII), and red blood cell distribution width-coefficient of variation (RDW-CV)] (*P* < 0.05). ROC, receiver operator characteristic; AUC, area under the curve; CI, confidence interval; LR, logistic regression; KNN, k-nearest neighbor; DT, decision tree; SVM, support vector machine; RF, and random forest; AdaBoost, adaptive boosting; GNB, Gaussian naive Bayes; WBC, white blood cell count; NLR, neutrophil-to-lymphocyte ratio; PLR, platelet to lymphocyte ratio; SII, systemic immune-inflammation index; RDW-CV, red blood cell distribution width-coefficient of variation.

All model discrimination performance results reported were from the best-performing models with optimized hyper parameters. As shown in Figures 3A,B and Table 4, our model had relatively ideal discrimination performance with a mean AUC of 0.788 (95% CI, 0.736–0.838), sensitivity of 0.741, specificity of 0.709, ACC of 0.715, precision of 0.358, and *F*1-score of 0.479. DeLong’s test revealed that our model’s AUC was significantly higher than that of the other models (*P* < 0.05). Furthermore, in terms of sensitivity, specificity, ACC, precision, and *F*1-score, our model outperformed all other models. Overall, our model had better discrimination performance than other models.

**TABLE 4:** Discrimination performance of all models and indices.

	AUC (95% CI)	SEN	SPE	ACC	Precision	*F*1-score	Brier	*P*-value
Our	0.788 (0.736–0.838)	0.741	0.709	0.715	0.358	0.479	0.187	
LR	0.721 (0.659–0.780)	0.672	0.646	0.651	0.293	0.406	0.222	**0.022**
KNN	0.632 (0.568–0.698)	0.616	0.594	0.598	0.247	0.351	0.249	**<0.001**
DT	0.673 (0.605–0.739)	0.693	0.586	0.608	0.267	0.385	0.235	**0.006**
SVM	0.726 (0.665–0.782)	0.683	0.629	0.639	0.287	0.402	0.219	**0.013**
RF	0.718 (0.659–0.777)	0.69	0.613	0.627	0.28	0.397	0.223	**0.004**
AdaBoost	0.714 (0.654–0.774)	0.651	0.666	0.663	0.298	0.407	0.239	**0.009**
GNB	0.691 (0.626–0.752)	0.687	0.609	0.62	0.282	0.394	0.281	**0.005**
WBC	0.590 (0.514–0.664)	0.54	0.625	0.611	0.239	0.329	0.254	**0.005**
NLR	0.619 (0.550–0.686)	0.633	0.568	0.579	0.241	0.347	0.253	**0.001**
PLR	0.584 (0.510–0.656)	0.607	0.533	0.546	0.219	0.321	0.263	**<0.001**
SII	0.589 (0.519–0.661)	0.548	0.625	0.611	0.241	0.333	0.259	**<0.001**
RDW-CV	0.645 (0.577–0.711)	0.614	0.62	0.621	0.259	0.364	0.242	**<0.001**

The best results are indicated underlined. *P* ≤ 0.05 are indicated in bold.

AUC, area under the curve; CI, confidence interval; SEN, sensitivity; SPE, specificity; ACC, accuracy; LR, logistic regression; KNN, k-nearest neighbor; DT, decision tree; SVM, support vector machine; RF, and random forest; AdaBoost, adaptive boosting; GNB, Gaussian naive Bayes; WBC, white blood cell count; NLR, neutrophil-to-lymphocyte ratio; PLR, platelet to lymphocyte ratio; SII, systemic immune-inflammation index; RDW-CV, red blood cell distribution width-coefficient of variation.

Our model’s discrimination performance was also superior to that of some well-studied CBC w/diff-derived clinical indices, such as WBC, RDW-CV, NLR, PLR, and SII. As shown in Figure 3C and Table 4, our model’s AUC was significantly higher than that of other indices (*P* < 0.05). In addition, when sensitivity, specificity, ACC, precision, and *F*1-score were all considered, our model outperformed all other clinical indices.

### The calibration performance of our model

**FIGURE 4:**
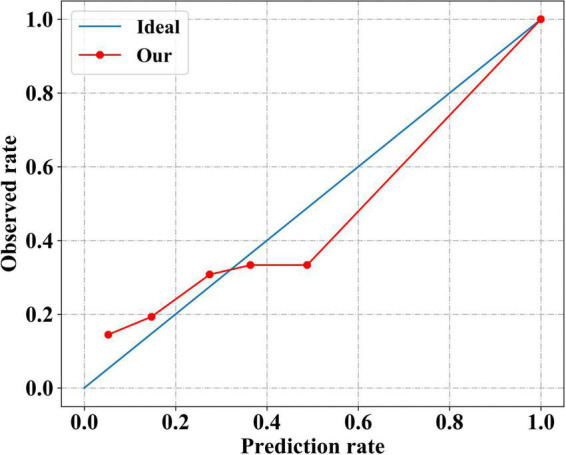
Calibration performance of our neural network model. The solid red line indicates the calibration curve of our neural network model, which closely matches the blue ideal calibration curve.

The calibration curve of our model was plotted to evaluate the calibration performance (Figure 4). The death risk predicted by our model agreed with the observed death rate to some extent, indicating that our model performed well in estimating the absolute risk. Intuitively, among patients with an actual mortality risk of less than 30%, our model slightly underestimated the mortality risk. In contrast, among patients with a higher actual mortality risk, our model overestimated the mortality risk slightly. As a more comprehensive assessment index of model performance, the Brier scores are shown in Table 4. Our model had a small Brier score, indicating it had good performance in both discrimination and calibration.

### Feature importance

**FIGURE 5:**
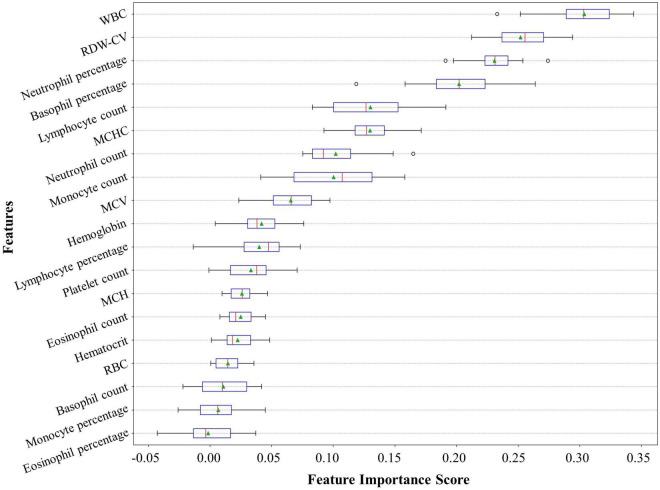
Feature importance ranking of our neural network model. The feature importance rank denotes how heavily our model relies on a predictor variable. The relative importance rank of the 19 predictor variables from the complete blood count with differential (CBC w/diff) test in our model is shown. CBC w/diff, complete blood count with differential; WBC, white blood cell; RDW-CV, red cell distribution width-coefficient of variation; MCHC, mean corpuscular hemoglobin concentration; MCV, mean corpuscular volume; MCH, mean corpuscular hemoglobin; RBC, red blood cell.

These predictors ranked in descending order of importance were WBC, RDW-CV, neutrophil percentage, basophil percentage, lymphocyte count, mean corpuscular hemoglobin concentration (MCHC), neutrophil count, monocyte count, mean corpuscular volume (MCV), hemoglobin, lymphocyte percentage, PLT, mean corpuscular hemoglobin (MCH), eosinophil count, hematocrit, red blood cell count (RBC), basophil count, monocyte percentage, and eosinophil percentage (Figure 5).

It can be seen that some variables played weak roles in improving the discrimination performance of the model. So, we dropped one to *N* variables with poor prediction values step by step and observed the change in the discrimination performance of our model. As shown in Table 5, we can see that: (1) dropping several variables with poor prediction values could help the model to improve its discrimination performance; (2) when seven variables were dropped, the discrimination performance of the model was the largest with an AUC of 0.812 (95% CI, 0.762–0.859) (*P* < 0.05); (3) the AUC of our model was decreasing significantly as more than ten variables were dropped.

**TABLE 5:** Discrimination performance of our model after dropping variables with poor prediction values.

	AUC (95% CI)	SEN	SPE	ACC	Precision	*F*1-score	*P*-value
drop_0	0.788 (0.736–0.838)	0.741	0.709	0.715	0.358	0.479	
drop_1	0.790 (0.737–0.839)	0.772	0.684	0.700	0.348	0.477	0.156
drop_2	0.789 (0.736–0.841)	0.768	0.675	0.693	0.338	0.469	0.195
drop_3	0.792 (0.740–0.842)	0.765	0.689	0.704	0.347	0.477	0.137
drop_4	0.792 (0.738–0.841)	0.766	0.703	0.713	0.360	0.486	0.129
drop_5	0.796 (0.744–0.845)	0.755	0.717	0.725	0.369	0.494	**0.025**
drop_6	0.797 (0.745–0.847)	0.743	0.720	0.723	0.367	0.488	**0.023**
drop_7	0.812 (0.762–0.859)	0.797	0.710	0.726	0.385	0.514	**0.012**
drop_8	0.806 (0.751–0.851)	0.768	0.708	0.718	0.375	0.497	**0.016**
drop_9	0.803 (0.749–0.849)	0.766	0.709	0.718	0.370	0.493	**0.017**
drop_10	0.782 (0.731–0.833)	0.737	0.686	0.696	0.337	0.461	0.161
drop_11	0.774 (0.721–0.825)	0.726	0.707	0.709	0.352	0.471	**0.019**
drop_12	0.770 (0.716–0.822)	0.708	0.702	0.703	0.340	0.458	**0.017**
drop_13	0.766 (0.710–0.819)	0.706	0.699	0.700	0.342	0.457	**0.014**
drop_14	0.720 (0.661–0.777)	0.713	0.653	0.664	0.309	0.429	**0.004**
drop_15	0.722 (0.662–0.780)	0.689	0.664	0.667	0.310	0.424	**0.005**
drop_16	0.719 (0.658–0.776)	0.702	0.665	0.671	0.311	0.430	**0.003**
drop_17	0.700 (0.635–0.760)	0.698	0.644	0.653	0.298	0.416	**0.003**
drop_18	0.623 (0.555–0.690)	0.588	0.618	0.613	0.250	0.350	**0.001**

The best results are indicated underlined. *P* ≤ 0.05 are indicated in bold. The “drop_*N*” means that dropping the last N variables in the feature importance ranking.

AUC, area under the curve; CI, confidence interval; SEN, sensitivity; SPE, specificity; ACC, accuracy.

## Discussion

Early clinical decision-making is crucial for patients suffering from severe AMI. Thus, in the present study, we developed a convenient and rapid risk stratification model for in-hospital mortality. This model consisted of 19 predictor variables from the CBC w/diff test and was built with a neural network algorithm. The discrimination performance of the model was superior to that of clinical indices derived from the CBC w/diff test and models built based on seven other classical machine learning algorithms. Moreover, as shown in the calibration curve, the model exhibited good performance in estimating the absolute risk. Finally, after using a permutation test to rank the 19 predictor variables in order of importance for the model, the top three important predictors were WBC, RDW-CV, and neutrophil percentage. Then, by dropping several variables with poor prediction values, we surprisingly got better discrimination performance of the model.

To further explore the clinical application of our model, we examined its performance in subgroups with different myocardial infarction types and demographics. We extracted subgroups of the ST-elevation myocardial infarction (STEMI) and the non-ST-elevation myocardial infarction (NSTEMI) patients according to the ICD codes from the discharge diagnosis list. Of the total 2,231 patients, 319 had a definite diagnosis of STEMI, and 442 had a definite diagnosis of NSTMI. After predicting the in-hospital mortality risk for two subgroups separately, we found that our model outperformed other clinical indicators in both subgroups (Supplementary Tables 1, 2). Additionally, as shown in Supplementary Table 3, our model performed well in both male and female subgroups, as well as in young (≤65 years) and old (>65 years) subgroups.

### Prognostic value of complete blood count with differential indices in acute myocardial infarction

Complete blood count with differential is commonly performed today because of its low cost and ease of availability in clinical practice. In recent years, the CBC w/diff indices have piqued the interest of researchers since they have been verified to provide a wealth of independent information on pathophysiology and risk stratification. The red blood cell indices provide information about the oxygen-carrying capacity. Elevated RDW (12) and decreased MCHC (23) have been linked to higher in-hospital and long-term mortality in patients with AMI. The white blood cell indices provide information about the inflammatory and immune systems. Leukocytes play important roles in the development and progression of AMI because they not only permeate the endothelial layer and induce formation of microvessels in the tunica intima, resulting in plaque rupture (24), but they also amplify the inflammatory cascade after AMI (25). The elevated WBC count was found to be connected to congestive heart failure, shock, and increased mortality among patients with AMI (8, 26). Furthermore, the prognostic value of the count and percentage of neutrophils in AMI has also been confirmed (27, 28). Platelet indices indicate thrombotic risk and inflammatory activation to some extent, since platelets participate in thrombogenesis and interact with leukocytes (29). Mean platelet volume (MPV) and platelet distribution width (PDW) are important markers of platelet activation (30), as well as strong and independent predictors of mortality in patients with AMI (31, 32). Given that oxygen-carrying capacity, inflammatory and immune status, and thrombotic risk all play key roles or may have interacted in the pathophysiology of AMI, several composite indices were developed and proved to be strong predictors of adverse outcomes following AMI. These composite indices include the NLR (9), PLR (33), WBC/MPV ratio (34, 35), and SII (11). The newly developed model in this study integrated all available indices in the CBC w/diff test reported by the MIMIC-IV database, and was supposed to have more substantial predictive power than other indices. In contrast to other machine-learning models and clinical indices, the excellent discrimination performance of our model was ultimately confirmed.

We ranked the predictors using a permutation test. The top three predictors were WBC, RDW-CV, and neutrophil percentage. The prognostic value and pathophysiological significance of these three predictors have been well studied, which partly confirmed the practical application value of our model. However, it is important to note that the high performance of the neural network-based algorithm comes at the sacrifice of the interpretability of the relationship between predictive factors and the outcome of interest. Although some blood components seem to have poor prediction values, which does not mean that they are of poor pathophysiological significance. The rank result can only be a hint, but not the evidence.

In this study, for the purpose of aiding in early clinical decision-making, we used the first CBC w/diff test result immediately after admission to create our model. Nevertheless, there is indisputable that the dynamic change of the blood cells has significant prognostic value in AMI. Foy et al. (36) have identified a universal recovery trajectory defined by exponential WBC decay and delayed linear growth of PLT, which provides a generic approach for identifying high-risk patients with acute inflammatory diseases, including AMI. It means that the CBC w/diff test has more prognostic value to be developed in the future.

### Machine learning algorithms in the prediction model

Most traditional risk models for cardiovascular disease are based on regression methods. While robust and useful, these methods are limited to using a small number of predictors and presupposing the linear and homogeneous effects of the predictors on the outcome. New methods for developing risk models are urgently needed because of the increasing ubiquity of large datasets, such as Electronic Health Records. Since machine learning algorithmic models can include more variables and produce more flexible relationships between predictors and outcomes, they have shown significant value in risk model development. For patients with AMI, state-of-the-art machine learning models have steadily improved the discrimination performance of risk stratification (3, 4, 37).

As canonical “black box” algorithms, neural networks apply the non-linear transformation to the predictor variables, hence being able to model many heterogeneous and non-linear effects (3). However, due to those multiple hidden layers in the transformation process, the interpretability of causal relationships between predictor variables and outcomes of interest can be challenging. Of note, interpretability is not necessary for the development of prediction models, where the focus is on the prediction performance instead of the predictors.

### Strengths and limitations

There are several strengths of the newly developed model. First, it is a well-performing CBC w/diff-based neural network model derived from a real-world cohort aiming at risk stratification purposes among patients with AMI in the CCU, making it suitable for severely ill patients diagnosed with AMI in the CCU. Of note, this model needs to be recalibrated and updated appropriately when used in other medical centers, which means that the hyper-parameters need to be slightly optimized. Second, the CBC w/diff test’s low cost and easy availability promotes clinical application and facilitates rapid clinical decision-making. Third, as intuitively demonstrated by the calibration curve, the new model slightly underestimated the mortality risk among patients with an actual mortality risk of less than 30% and overestimated the mortality risk among patients with a higher actual mortality risk, thereby avoiding unnecessary and excessive tests in low-risk patients, and, more importantly, avoiding treatment delays in high-risk patients. Fourth, the neural network algorithm used in the new model showed competence in prediction and can be applied to develop other prediction models.

However, the new model has several limitations. First, the retrospective study design makes the data susceptible to selection and measurement biases. Yet, the cohort analyzed in this study was collected prospectively and reflected real-world data, which may convey more practical significance. Second, causal relationships between predictors and the outcome cannot be established due to limited interpretability. This is a commonly recognized shortcoming of neural network models. All causal inferences require further experimental verification. Third, the model has not been externally validated. However, iterated cross-validation enhances the strength of the results. Fourth, missing data may lead to potential bias. But, the proportion of missing values was very low (less than 7.4%) in the study cohort. Moreover, for missing data imputation, we adopted the missForest method, an excellent machine learning-based data imputation algorithm. Fifth, the predictor variable list lacks platelet indices, including PDW and MPV, as they are unavailable in the database. Finally, although the newly developed CBC w/diff-based model has excellent performance in predicting in-hospital mortality among patients with AMI in the CCU, it is obvious that the comprehensive risk of these patients cannot be assessed solely based on this laboratory test. The additional value of the CBC w/diff variables to other existing risk scores, such as the thrombolysis in myocardial infarction (TIMI) or global registry of acute coronary events (GRACE) scores, was not determined, as most components of these scores were not available in this public database. According to the original publication of the GRACE model (38), it’s AUC in the derivation, the internal validation, and the external validation datasets were 0.83, 0.85, and 0.79, respectively. It seems that the predictive ACC of the CBC w/diff-based model is close to that of the GRACE model. In the future, this model should strive to validate its prognostic value in the combined analysis with conventional scores, such as the GRACE and TIMI scores.

## Conclusion

We developed a risk prediction model based on the neural network algorithm for in-hospital mortality among patients with AMI in the CCU. The top three important predictors in this model were WBC, RDW-CV, and neutrophil percentage. The model is simple and easy to use. After entering 19 variables from the CBC w/diff test, the final prediction result can be easily obtained with just one click on a Python command. The relevant codes are available on the GitHub website.^3^ We believe the proposed method can be used as a quick and easy risk assessment tool for clinical decision-making.

## Data availability statement

Publicly available datasets were analyzed in this study. This data can be found here: MIMIC-IV repository, https://doi.org/10.13026/s6n6-xd98.

## Ethics statement

Ethical review and approval was not required for this study on human participants in accordance with the local legislation and institutional requirements. Written informed consent for participation was not required for this study in accordance with the national legislation and the institutional requirements.

## Author contributions

YW, CFL, DG, and FR: conceptualization and project administration. CFL, MY, and BR: data curation and analysis. CL, JZ, and ZL: methodology. YW and CFL: writing and editing. All authors read and approved the final manuscript.

## Footnotes

1
https://doi.org/10.13026/s6n6-xd98

2
https://github.com/MIT-LCP/mimic-code

3
https://github.com/duweidai/CBC2AMI

## Abbreviations

ACC: accuracy
AMI: acute myocardial infarction
AdaBoost: adaptive boosting
AUC: area under the curve
BIDMC: Beth Israel Deaconess Medical Center
BMI: body mass index
CBC w/diff: complete blood count with differential
CKMB: creatine kinase - MB isoenzyme
CI: confidence interval
CCU: coronary care unit
DBP: diastolic blood pressure
DT: decision tree
FC: fully connected layer
GNB: Gaussian naive Bayes
GRACE: Global registry of acute coronary events
ICD: International classification of diseases
KNN: k-nearest neighbor
LR: logistic regression
MCH: mean corpuscular hemoglobin
MCHC: mean corpuscular hemoglobin concentration
MCV: mean corpuscular volume
MPV: mean platelet volume
MIMIC: Medical Information Mart for Intensive Care
NLR: neutrophil-to-lymphocyte ratio
NSTEMI: non-ST-elevation myocardial infarction
PDW: platelet distribution width
PLR: platelet to lymphocyte ratio
RF: random forest
RBC: red blood cell
ROC: receiver operating characteristic
ReLU: rectified linear unit
RDW-CV: red blood cell distribution width-coefficient of variation
SBP: systolic blood pressure
SEN: sensitivity
SII: systemic immune-inflammation index
SPE: specificity
SpO_2_: pulse oxygen saturation
SQL: structure query language
STEMI: ST-elevation myocardial infarction
SVM: support vector machine
TIMI: thrombolysis in myocardial infarction
TRIPOD: transparent reporting of multivariable prediction model for individual prognosis or diagnosis
WBC: white blood cell count.

## References

[1] Rahimi K Duncan M Pitcher A Emdin CA Goldacre MJ. Mortality from heart failure, acute myocardial infarction and other ischaemic heart disease in England and Oxford: a trend study of multiple-cause-coded death certification. *J Epidemiol Community Health.* (2015) 69:1000–5. 10.1136/jech-2015-205689 26136081 PMC4602272

[2] de Vreede JJ Gorgels AP Verstraaten GM Vermeer F Dassen WR Wellens HJ. Did prognosis after acute myocardial infarction change during the past 30 years? A meta-analysis. *J Am Coll Cardiol.* (1991) 18:698–706. 10.1016/0735-1097(91)90792-8 1831213

[3] Goldstein BA Navar AM Carter RE. Moving beyond regression techniques in cardiovascular risk prediction: applying machine learning to address analytic challenges. *Eur Heart J.* (2017) 38:1805–14. 10.1093/eurheartj/ehw302 27436868 PMC5837244

[4] Kwon JM Jeon KH Kim HM Kim MJ Lim S Kim KH Deep-learning-based risk stratification for mortality of patients with acute myocardial infarction. *PLoS One.* (2019) 14:e0224502. 10.1371/journal.pone.0224502 31671144 PMC6822714

[5] Fox KA Dabbous OH Goldberg RJ Pieper KS Eagle KA Van de Werf F Prediction of risk of death and myocardial infarction in the six months after presentation with acute coronary syndrome: prospective multinational observational study (GRACE). *BMJ.* (2006) 333:1091. 10.1136/bmj.38985.646481.55 17032691 PMC1661748

[6] Addala S Grines CL Dixon SR Stone GW Boura JA Ochoa AB Predicting mortality in patients with ST-elevation myocardial infarction treated with primary percutaneous coronary intervention (PAMI risk score). *Am J Cardiol.* (2004) 93:629–32. 10.1016/j.amjcard.2003.11.036 14996596

[7] Morrow DA Antman EM Charlesworth A Cairns R Murphy SA de Lemos JA TIMI risk score for ST-elevation myocardial infarction: a convenient, bedside, clinical score for risk assessment at presentation: an intravenous NPA for treatment of infarcting myocardium early II trial substudy. *Circulation.* (2000) 102:2031–7. 10.1161/01.cir.102.17.2031 11044416

[8] Sabatine MS Morrow DA Cannon CP Murphy SA Demopoulos LA DiBattiste PM Relationship between baseline white blood cell count and degree of coronary artery disease and mortality in patients with acute coronary syndromes: a tactics-TIMI 18 (treat angina with aggrastat and determine cost of therapy with an invasive or conservative strategy- thrombolysis in myocardial infarction 18 trial)substudy. *J Am Coll Cardiol.* (2002) 40:1761–8. 10.1016/s0735-1097(02)02484-1 12446059

[9] Azab B Zaher M Weiserbs KF Torbey E Lacossiere K Gaddam S Usefulness of neutrophil to lymphocyte ratio in predicting short- and long-term mortality after non-ST-elevation myocardial infarction. *Am J Cardiol.* (2010) 106:470–6. 10.1016/j.amjcard.2010.03.062 20691303

[10] Azab B Shah N Akerman M McGinn JT Jr. Value of platelet/lymphocyte ratio as a predictor of all-cause mortality after non-ST-elevation myocardial infarction. *J Thromb Thrombolysis.* (2012) 34:326–34. 10.1007/s11239-012-0718-6 22466812

[11] Öcal L Keskin M Cerşit S Eren H Özgün Çakmak E Karagöz A Systemic immune-inflammation index predicts in-hospital and long-term outcomes in patients with ST-segment elevation myocardial infarction. *Coron Artery Dis.* (2022) 33:251–60. 10.1097/mca.0000000000001117 35044330

[12] Dabbah S Hammerman H Markiewicz W Aronson D. Relation between red cell distribution width and clinical outcomes after acute myocardial infarction. *Am J Cardiol.* (2010) 105:312–7. 10.1016/j.amjcard.2009.09.027 20102941

[13] Goldberger AL Amaral LA Glass L Hausdorff JM Ivanov PC Mark RG Physiobank, physiotoolkit, and physionet: components of a new research resource for complex physiologic signals. *Circulation.* (2000) 101:E215–20. 10.1161/01.cir.101.23.e215 10851218

[14] Collins GS Reitsma JB Altman DG Moons KG. Transparent reporting of a multivariable prediction model for individual prognosis or diagnosis (TRIPOD): the TRIPOD statement. The TRIPOD group. *Circulation.* (2015) 131:211–9. 10.1161/circulationaha.114.014508 25561516 PMC4297220

[15] Stekhoven DJ Bühlmann P. Missforest–non-parametric missing value imputation for mixed-type data. *Bioinformatics.* (2012) 28:112–8. 10.1093/bioinformatics/btr597 22039212

[16] Nair V Hinton GE editors. Rectified linear units improve restricted boltzmann machines Vinod Nair. In: *Proceedings of the International Conference on International Conference on Machine Learning.* Haifa (2010).

[17] Srivastava N Hinton G Krizhevsky A Sutskever I Salakhutdinov R. Dropout: a simple way to prevent neural networks from overfitting. *J Machine Learn Res.* (2014) 15:1929–58. 10.1109/TCYB.2020.3035282 33259321

[18] Han J Moraga C editors. The influence of the sigmoid function parameters on the speed of backpropagation learning. In: *Proceedings of the International Workshop on Artificial Neural Networks: From Natural to Artificial Neural Computation.* Torremolinos (1995).

[19] Amari S. Backpropagation and stochastic gradient descent method. *Neurocomputing.* (1993) 5:185–96. 10.1016/0925-2312(93)90006-O

[20] Ho Y Wookey S. The real-world-weight cross-entropy loss function: modeling the costs of mislabeling. *IEEE Access.* (2020) 8:4806–13. 10.1109/ACCESS.2019.2962617

[21] Alba AC Agoritsas T Walsh M Hanna S Iorio A Devereaux PJ Discrimination and calibration of clinical prediction models: users’ guides to the medical literature. *JAMA.* (2017) 318:1377–84. 10.1001/jama.2017.12126 29049590

[22] Ojala M Garriga GC. Permutation tests for studying classifier performance. *J Machine Learn Res.* (2010) 11:1833–63.

[23] Huang YL Hu ZD. Lower mean corpuscular hemoglobin concentration is associated with poorer outcomes in intensive care unit admitted patients with acute myocardial infarction. *Ann Transl Med.* (2016) 4:190. 10.21037/atm.2016.03.42 27294086 PMC4885905

[24] Madjid M Awan I Willerson JT Casscells SW. Leukocyte count and coronary heart disease: implications for risk assessment. *J Am Coll Cardiol.* (2004) 44:1945–56. 10.1016/j.jacc.2004.07.056 15542275

[25] Yan X Anzai A Katsumata Y Matsuhashi T Ito K Endo J Temporal dynamics of cardiac immune cell accumulation following acute myocardial infarction. *J Mol Cell Cardiol.* (2013) 62:24–35. 10.1016/j.yjmcc.2013.04.023 23644221

[26] Barron HV Cannon CP Murphy SA Braunwald E Gibson CM. Association between white blood cell count, epicardial blood flow, myocardial perfusion, and clinical outcomes in the setting of acute myocardial infarction: a thrombolysis in myocardial infarction 10 substudy. *Circulation.* (2000) 102:2329–34. 10.1161/01.cir.102.19.2329 11067784

[27] Meissner J Irfan A Twerenbold R Mueller S Reiter M Haaf P Use of neutrophil count in early diagnosis and risk stratification of AMI. *Am J Med.* (2011) 124:534–42. 10.1016/j.amjmed.2010.10.023 21507368

[28] Men M Zhang L Li T Mi B Wang T Fan Y Prognostic value of the percentage of neutrophils on admission in patients with ST-elevated myocardial infarction undergoing primary percutaneous coronary intervention. *Arch Med Res.* (2015) 46:274–9. 10.1016/j.arcmed.2015.05.002 25989351

[29] Croce K Libby P. Intertwining of thrombosis and inflammation in atherosclerosis. *Curr Opin Hematol.* (2007) 14:55–61. 10.1097/00062752-200701000-00011 17133101

[30] Kim YG Suh JW Yoon CH Oh IY Cho YS Youn TJ Platelet volume indices are associated with high residual platelet reactivity after antiplatelet therapy in patients undergoing percutaneous coronary intervention. *J Atheroscler Thromb.* (2014) 21:445–53. 10.5551/jat.20156 24430786

[31] Huczek Z Kochman J Filipiak KJ Horszczaruk GJ Grabowski M Piatkowski R Mean platelet volume on admission predicts impaired reperfusion and long-term mortality in acute myocardial infarction treated with primary percutaneous coronary intervention. *J Am Coll Cardiol.* (2005) 46:284–90. 10.1016/j.jacc.2005.03.065 16022956

[32] Rechciński T Jasińska A Foryś J Krzemińska-Pakuła M Wierzbowska-Drabik K Plewka M Prognostic value of platelet indices after acute myocardial infarction treated with primary percutaneous coronary intervention. *Cardiol J.* (2013) 20:491–8. 10.5603/cj.2013.0134 24469872

[33] Li L Ma Y Geng XB Tan Z Wang JH Cui C Platelet-to-lymphocyte ratio relates to poor prognosis in elderly patients with acute myocardial infarction. *Aging Clin Exp Res.* (2021) 33:619–24. 10.1007/s40520-020-01555-7 32301030

[34] Dehghani MR Rezaei Y Taghipour-Sani L. White blood cell count to mean platelet volume ratio as a novel non-invasive marker predicting long-term outcomes in patients with non-ST elevation acute coronary syndrome. *Cardiol J.* (2015) 22:437–45. 10.5603/CJ.a2015.0015 25733319

[35] Çiçek G Açıkgöz SK Yayla Ç Kundi H İleri M. White blood cell count to mean platelet volume ratio: a novel and promising prognostic marker for ST-segment elevation myocardial infarction. *Cardiol J.* (2016) 23:225–35. 10.5603/CJ.a2016.0001 26779969

[36] Foy BH Sundt TM Carlson JCT Aguirre AD Higgins JM. Human acute inflammatory recovery is defined by co-regulatory dynamics of white blood cell and platelet populations. *Nat Commun.* (2022) 13:4705. 10.1038/s41467-022-32222-2 35995789 PMC9395541

[37] Matheny ME Ricket I Goodrich CA Shah RU Stabler ME Perkins AM Development of electronic health record-based prediction models for 30-day readmission risk among patients hospitalized for acute myocardial infarction. *JAMA Netw Open.* (2021) 4:e2035782. 10.1001/jamanetworkopen.2020.35782 33512518 PMC7846941

[38] Granger CB Goldberg RJ Dabbous O Pieper KS Eagle KA Cannon CP Predictors of hospital mortality in the global registry of acute coronary events. *Arch Intern Med.* (2003) 163:2345–53. 10.1001/archinte.163.19.2345 14581255

