# Supplementary material for: Development of a complete blood count with differential—based prediction model for in-hospital mortality among patients with acute myocardial infarction in the coronary care unit
Source: Front Cardiovasc Med. 2022 Oct 5;9:1001356. doi: 10.3389/fcvm.2022.1001356 (PMC9581274; doi:10.3389/fcvm.2022.1001356)
Supplement: Supplementary file 1 [file Data_Sheet_1.docx]

Table S1. Discrimination performance of our model and clinical indices among patients in the subgroup with STEMI.

| STEMI (n=319) | AUC (95% CI) | SEN | SPE | ACC | Precision | F1-score | Brier |
| --- | --- | --- | --- | --- | --- | --- | --- |
| Our | 0.832 (0.718-0.927) | 0.895 | 0.721 | 0.756 | 0.459 | 0.600 | 0.178 |
| WBC | 0.654 (0.460-0.828) | 0.563 | 0.690 | 0.670 | 0.337 | 0.408 | 0.253 |
| NLR | 0.617 (0.432-0.787) | 0.528 | 0.658 | 0.629 | 0.279 | 0.358 | 0.246 |
| PLR | 0.534 (0.321-0.745) | 0.471 | 0.628 | 0.597 | 0.257 | 0.320 | 0.259 |
| SII | 0.584 (0.385-0.771) | 0.456 | 0.700 | 0.644 | 0.297 | 0.336 | 0.254 |
| RDW-CV | 0.649 (0.458-0.822) | 0.570 | 0.671 | 0.648 | 0.309 | 0.393 | 0.239 |

The best results are indicated underlined.

STEMI, ST-elevation myocardial infarction; AUC, area under the curve; CI, confidence interval; SEN, sensitivity; SPE, specificity; ACC, accuracy; WBC, white blood cell count; NLR, neutrophil-to-lymphocyte ratio; PLR, platelet to lymphocyte ratio; SII, systemic immune-inflammation index; RDW-CV, red blood cell distribution width-coefficient of variation.

Table S2. Discrimination performance of our model and clinical indices among patients in the subgroup with NSTEMI.

| NSTEMI (n=442) | AUC (95% CI) | SEN | SPE | ACC | Precision | F1-score | Brier |
| --- | --- | --- | --- | --- | --- | --- | --- |
| Our | 0.824 (0.721-0.911) | 0.850 | 0.717 | 0.743 | 0.446 | 0.578 | 0.180 |
| WBC | 0.548 (0.392-0.699) | 0.525 | 0.588 | 0.571 | 0.248 | 0.330 | 0.258 |
| NLR | 0.594 (0.447-0.730) | 0.578 | 0.584 | 0.589 | 0.270 | 0.367 | 0.269 |
| PLR | 0.563 (0.399-0.723) | 0.466 | 0.672 | 0.634 | 0.276 | 0.344 | 0.265 |
| SII | 0.579 (0.426-0.723) | 0.549 | 0.592 | 0.586 | 0.272 | 0.360 | 0.270 |
| RDW-CV | 0.585 (0.431-0.736) | 0.503 | 0.680 | 0.646 | 0.299 | 0.362 | 0.247 |

The best results are indicated underlined.

NSTEMI, non-ST-elevation myocardial infarction; AUC, area under the curve; CI, confidence interval; SEN, sensitivity; SPE, specificity; ACC, accuracy; WBC, white blood cell count; NLR, neutrophil-to-lymphocyte ratio; PLR, platelet to lymphocyte ratio; SII, systemic immune-inflammation index; RDW-CV, red blood cell distribution width-coefficient of variation.

Table S3. Discrimination performance of our model among patients in subgroups with different demographics.

|  | AUC (95% CI) | SEN | SPE | ACC | Precision | F1-score | Brier |
| --- | --- | --- | --- | --- | --- | --- | --- |
| Male (n=1378) | 0.822 (0.759-0.878) | 0.812 | 0.718 | 0.734 | 0.375 | 0.506 | 0.178 |
| Female (n=853) | 0.823 (0.747-0.890) | 0.804 | 0.732 | 0.745 | 0.432 | 0.557 | 0.176 |
| Young (≤65y, n=657) | 0.895 (0.821-0.954) | 0.880 | 0.814 | 0.821 | 0.405 | 0.540 | 0.135 |
| Old (＞65y, n=1574) | 0.814 (0.757-0.866) | 0.799 | 0.698 | 0.719 | 0.411 | 0.539 | 0.177 |

AUC, area under the curve; CI, confidence interval; SEN, sensitivity; SPE, specificity; ACC, accuracy; WBC, white blood cell count; NLR, neutrophil-to-lymphocyte ratio; PLR, platelet to lymphocyte ratio; SII, systemic immune-inflammation index; RDW-CV, red blood cell distribution width-coefficient of variation.
